# CHIR99021 combined with retinoic acid promotes the differentiation of primordial germ cells from human embryonic stem cells

**DOI:** 10.18632/oncotarget.13958

**Published:** 2016-12-15

**Authors:** Tingting Cheng, Kui Zhai, Yan Chang, Guidong Yao, Jiahuan He, Fang Wang, Huijuan Kong, Hang Xin, Huiwen Wang, Meng Jin, Bing Gong, Lei Gu, Zhiguang Yang, Yanyun Wu, Guangju Ji, Yingpu Sun

**Affiliations:** ^1^ Center for Reproductive Medicine, The First Affiliated Hospital of Zhengzhou University, Zhengzhou, China; ^2^ National Laboratory of Biomacromolecules, Institute of Biophysics, Chinese Academy of Sciences, Beijing, China; ^3^ Department of Cardiac Surgery, State Key Laboratory of Cardiovascular Disease, Fuwai Hospital, National Center for Cardiovascular Disease, Chinese Academy of Medical Sciences and Peking Union Medical College, Beijing, China

**Keywords:** CHIR99021, retinoic acid, primordial germ cells, human embryonic stem cells, β-catenin

## Abstract

Primordial germ cells (PGCs) derived from human embryonic stem cells (hESCs) represent as a desirable experimental model as well as a potential strategy for treating male infertility. Here, we developed a simple and feasible method for differentiation of PGCs from hESCs by using CHIR99021 (an inhibitor of glycogen synthase kinase 3) and retinoic acid (RA). We firstly found that the deleted in azoospermia-like (DAZL) protein can be detected in 3 d CHIR99021 plus 9 d retinoic acid treated cultures and 12 d CHIR99021 plus retinoic acid co-treated cultures, but not expressed in single CHIR99021 treated cultures, single retinoic acid treated cultures, as well as 3 d retinoic acid plus 9 d CHIR99021 treated cultures. Next, we showed that several PGCs’ markers were expressed in the 12 d CHIR99021 and retinoic acid co-treated cultures or 3 d CHIR99021 plus 9 d retinoic acid treated cultures. Moreover, meiosis was initiated in CHIR99021 and retinoic acid co-treated cultures as evidenced by a significant expression of the punctate synaptonemal complex protein 3 (SCP3). Fluorescent *in situ* hybridization (FISH) analysis indicated that a small percentage of putative 1N populations were formed. Mechanically, we found that β-catenin relocated into nucleus after the treatment of 3 d CHIR99021 suggesting that Wnt signaling pathway was activated. Furthermore, blockade of Wnt signaling pathway by IWR-1 can reverse CHIR99021 and retinoic acid mediated-effects. Taken together, our results indicate that CHIR99021 combined with retinoic acid can effectively differentiate hESCs into PGCs via activating Wnt signaling pathway.

## INTRODUCTION

Infertility affects approximately 10–15% of couples, with male factors accounting for 40–60% of all the cases. Although advances in assisted reproductive technologies (ART) have allowed these patients to have their offspring, the pathogenesis of infertility especially male infertility, such as non-obstructive azoospermia, immune caused azoospermia, and unexplained azoospermia are still not fully explored. Germ cells have been used for studying spermatogenesis *in vitro* and pathological mechanisms of male infertility. So far, much progress has been made in the derivation of male differentiated germ cells from hESCs and induced pluripotent stem cells (iPSCs) [1–17]. Moreover, several growth factors, such as bone morphogenetic protein 4 (BMP4), BMP7, BMP8b, stem cell factor (SCF), epidermal growth factor (EGF), retinoic acid (RA) have been used in the differentiation of PGC-like cells (PGCLCs) from hESCs or iPSCs [2–6, 12, 15, 16, 18]. Besides, overexpression of the spermatogenesis-related genes including DAZL and VASA (also called DDX4) seems to be an another strategy [9, 11]. It is reported that male gametes derived from mESCs/iPSCs function to produce the viable offsprings suggesting that there are possibilities to get germ cells *in vitro* from hESCs and to provide potential cures for male infertility. [19, 20]. Thus, a comprehensive understanding of how to differentiate human male gametes is still urgent and necessary.

Spermatogenesis is an extremely complex process *in vivo*. It starts from spermatogonia to spermatozoa including a proliferative phase, a meiotic phase and a morphogenetic phase in most mammals [21]. Furthermore, the long-term production of billions of spermatozoa relies on the regulated proliferation and differentiation of spermatogonial stem cells (SSCs). The process of the transformation from SSCs into millions of haploid spermatozoa is elaborately organized in time and space [22].

Recently, the role of the Wnt/β-catenin signaling pathway in spermatogenesis has been widely discussed both in mammalian and non-mammalian vertebrate species [23–26]. However, whether it is involved in the derivation of PGCs from hESCs *in vitro* is unclear. It is well known that activation of the canonical Wnt/β-catenin signaling pathway by the Wnt ligand protein binds to Frizzled and Lrp5/6 receptors inhibits the GSK3β (glycogen synthase kinase 3β)-mediated degradation of β-catenin and results in the accumulation of cytoplasmic β-catenin. Then it translocates into the nucleus where it interacts with LEF/TCF transcriptional complexes to regulate the downstream target genes [27–29]. Here, we describe a crosstalk between CHIR99021 and RA in the differentiation of PGCs from hESCs via activating Wnt signaling pathway. CHIR99021 activated Wnt signaling pathway and then initiated the differentiation of hESCs by inhibiting the GSK3β mediated degradation of β-catenin; meanwhile, RA upregulated c-Kit. Those two compounds together promote the emergence of PGCs from hESCs. Our results highlight a simple and flexible method for inducing PGCs from hESCs that may provide a broad implication for studying male infertility.

## RESULTS

### CHIR99021 combined with RA induces the emergence of primitive DAZL-positive cells

**Figure 1:**
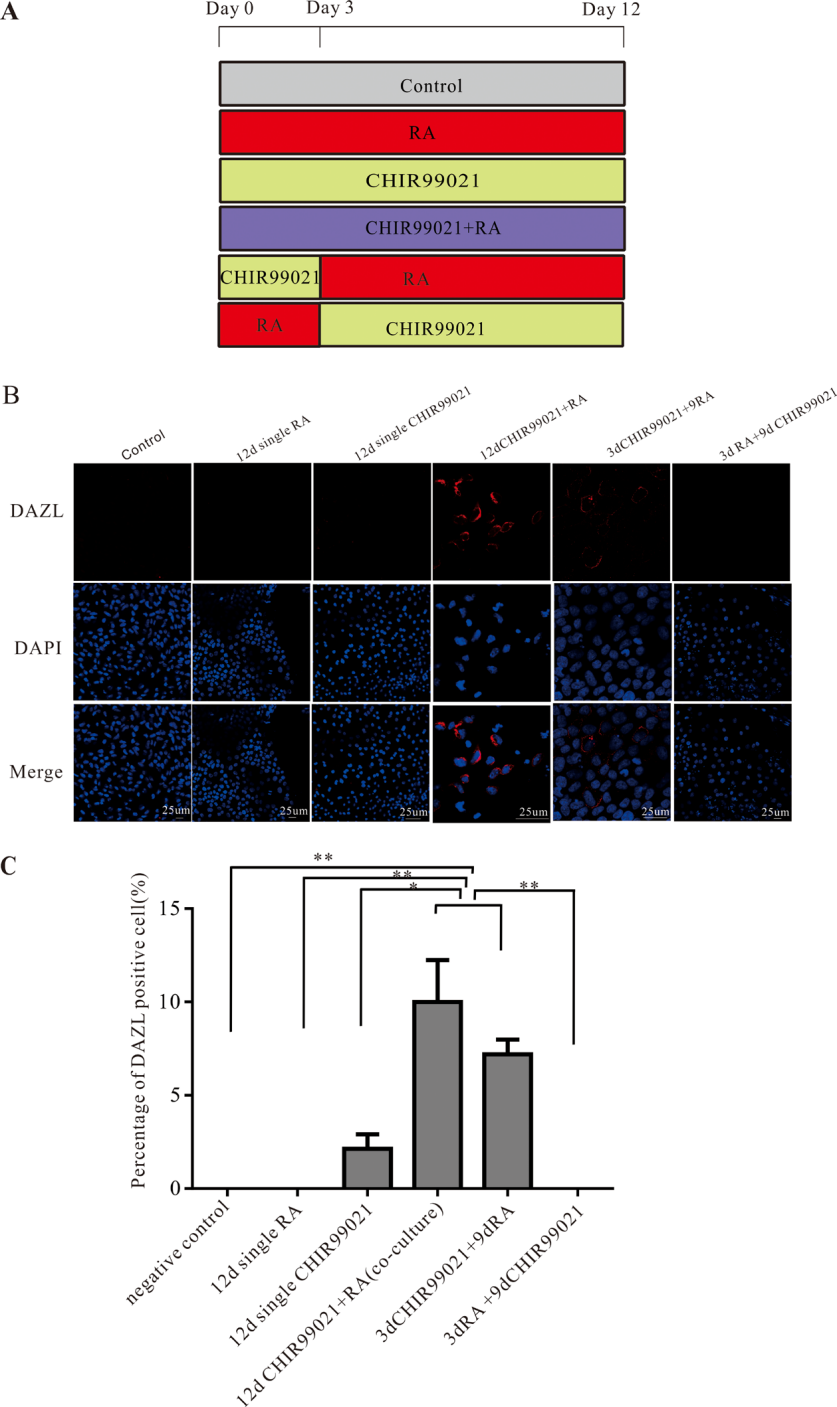
CHIR99021 combined with RA can induce the PGCs from hESCs (**A**) The protocol used for the differentiation of hESCs into PGCs. (**B**) The hESCs were treated by CHIR99021 and/or RA as indicated in (A). The DAZL positive cells were detected by using immunofluorescence staining nuclei were counterstained with DAPI. Scale bars, 25 μm. (**C**) Summary data showing the mean number of the DAZL positive cells in the different treatments. All data are presented as means ± SEM. *p < 0.05, **p < 0.01.

To access whether CHIR99021 and/or RA can induce PGCs from hESCs (Figure 1A), DAZL protein was initially detected by using immunofluorescence experiments. As shown in Figure 1B, among the different treatments, single CHIR99021, single RA and 3 d RA plus 9 d CHIR99021 did not induce the emergence of DAZL-positive cells. In contrast, DAZL protein was found to be expressed in cells treated by 3 d CHIR99021 plus 9 d RA and 12 d CHIR99021 plus RA (co-culture) groups. These DAZL-positive cells accounted for nearly 8–10% of the total cells. In addition, there were more DAZL-positive cells in the 12 d CHIR99021 plus RA (co-culture) treatment compared to the 3 d CHIR99021 plus 9 d RA treatment but without any significant difference (as shown in Figure 1C). Collectively, these results illustrate that CHIR99021 and RA can work together to induce PGCs from hESCs.

### DAZL-positive cells exhibit a phenotype comparable to that of migratory PGCs

**Figure 2:**
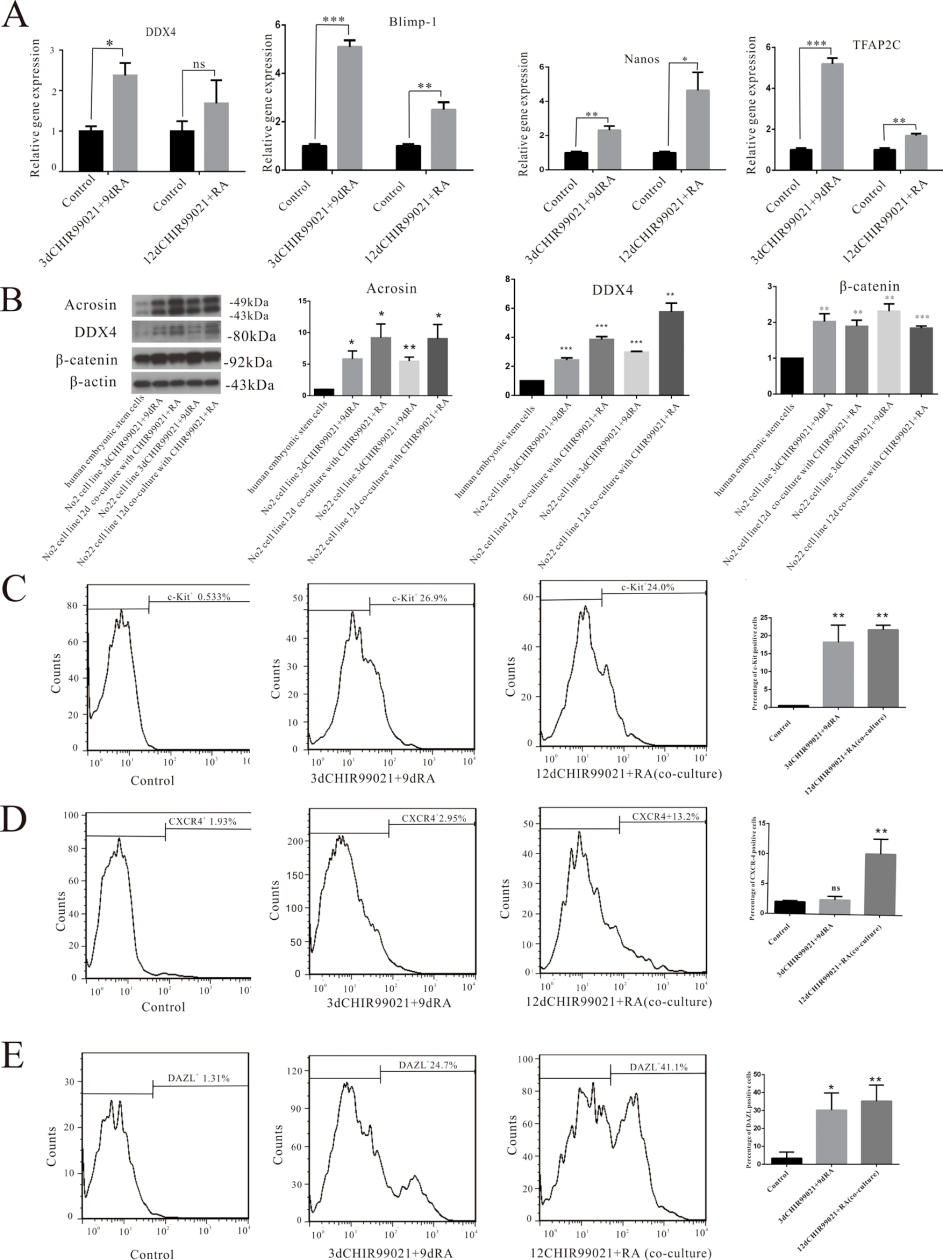
DAZL-positive cells have a phenotype comparable to that of migretory PGCs (**A**) mRNA levels of DDX4, Blimp-1, Nanos and TFAP2C was determined by RT-qPCR. The two hESCs lines were treated by 12 d CHIR99021 plus RA co-culture and 3 d CHIR99021 plus 9 d RA. The undifferentiated hESCs were used as control. (**B**) The two hESCs lines were treated by 12 d CHIR99021 plus RA co-culture or 3 d CHIR99021 plus 9 d RA. The undifferentiated hESCs were used as control. Then, Acrosin, DDX4, β-catenin and β-actin were detected by Immunoblots. All data are presented as means ± SEM in three independent experiments vs undifferentiated hESCs. (**C–E**) Flow cytometry analysis of c-Kit^+^, CXCR4^+^ and DAZL^+^ cells. The undifferentiated hESCs were used as a control shown on the left. The percentage shown in the histogram is the rate of positive cells. The middle icon represents 3 d CHIR99021 plus 9 d RA, the right icon stands for 12 d CHIR99021 and RA co-culture treatment. All data are presented as means ± SEM in three independent experiments. *p < 0.05, **p < 0.01, ***p < 0.001.

To explore the phenotype of these induced cells, several markers of PGCs such as DDX4, Blimp-1, Nanos, and TFAP2C were measured in our experimental conditions. As shown in Figure 2A, we found that the mRNA levels of several PGC markers including DDX4, Blimp-1, Nanos and TFAP2C were significantly up-regulated in the 3 d CHIR99021 plus 9 d RA and 12 d CHIR99021 plus RA (co-culture) groups. Besides, in contrast to the undifferentiated hESCs, the expression levels of acrosin and DDX4 were significantly higher in both lines. Furthermore, β-catenin had a significantly increased expression compared with the undifferentiated group (Figure 2B).

Flow cytometry analysis was implemented to further determine the phenotype of these induced cells. c-Kit, a critical cell surface marker that distinguishes differentiated spermatogonia (also called Kit), together with RA, can direct the differentiation of spermatogonia throughout the male reproductive lifespan [30]. We observed the frequencies of c-Kit positive cells in the 3 d CHIR99021 plus 9 d RA and 12 d CHIR99021 plus RA (co-culture) groups were 26.9% and 24%, respectively (Figure 2C). Here, we used the chemokine receptor CXCR4 as a positive marker, given that CXCR4 receptor is mainly expressed on the migrating germ cells [31], we found that CXCR4 positive cells account for approximately 13.2% in 12 d CHIR99021 plus RA (co-culture) group. However, the percentage of CXCR4 positive cells in 3 d CHIR99021 plus 9 d RA are lower than the co-culture group and just account for 2.95% which may explained as migrating germ cells was less in this induced process(Figure 2D). Furthermore, the percentage of DAZL positive cells in the 3 d CHIR99021 plus 9 d RA and 12 d CHIR99021 plus RA (co-culture) groups were 24.7% and 41.1%, respectively (Figure 2E). Taken together, these results further confirmed that CHIR99021 combined with RA can effectively differentiate hESCs into PGCs.

### Putative haploid cells were formed in CHIR99021 and RA treated cultures

**Figure 3:**
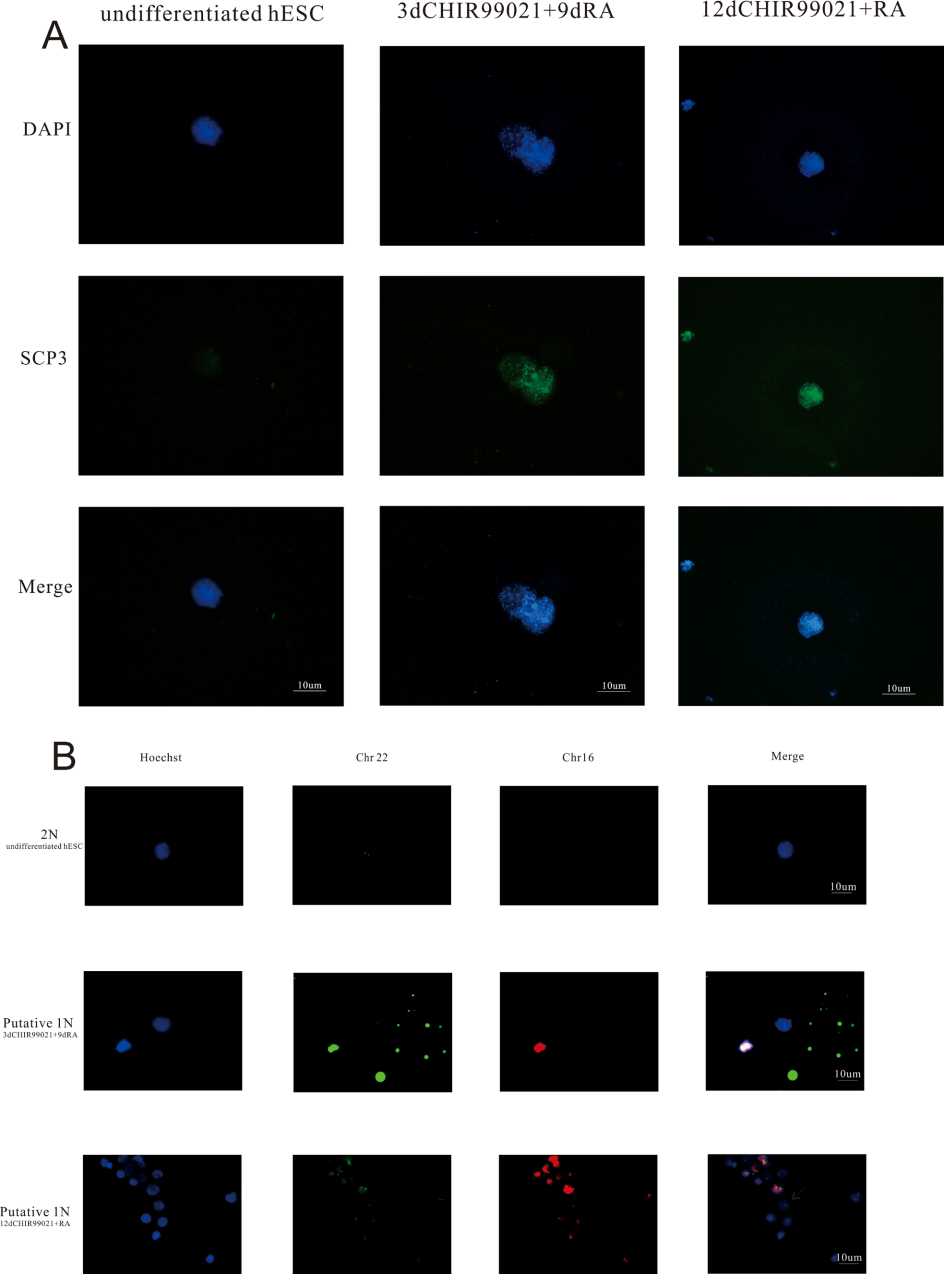
Putative haploid cells were formed in this induced process (**A**) Immunofluorescence staining with SCP3 is shown. Scale bars, 10 μm. (**B**) FISH with probe against autosomal chromosome 16 and chromosome 22 in the undifferentiated hESCs group and two induced groups. Scale bars, 10 μm.

Previous studies showed that post-meiotic germ cells can be obtained from hESCs [1, 11, 32]. We next determined whether meiotic was happened and even haploid cells were produced. Here, we used SCP3 which is indicative biomarker of synaptonemal complex formation in meiotic prophase I to examine the meiotic progress in our experiments [33]. As shown in Figure 3A, compared with undifferentiated hESCs group, punctate pattern was significantly increased in both induced groups indicating that the initiation of meiosis starts. Furthermore, FISH analysis was carried out to verify whether haploid cells were produced. As shown in Figure 3B, two probes for chromosome 16 and 22 were used. As expected, putative 1N cells possessed a single chromosome 16 and 22, whereas undifferentiated hESCs (2N) carried 2 chromosomes. These results indicate that human primordial germ cells differentiated from hESCs could even possess the ability to enter into the meiosis and form the haploid cells.

### Wnt signaling pathway was involved in the initiation of the differentiation of hESCs into PGCs

**Figure 4:**
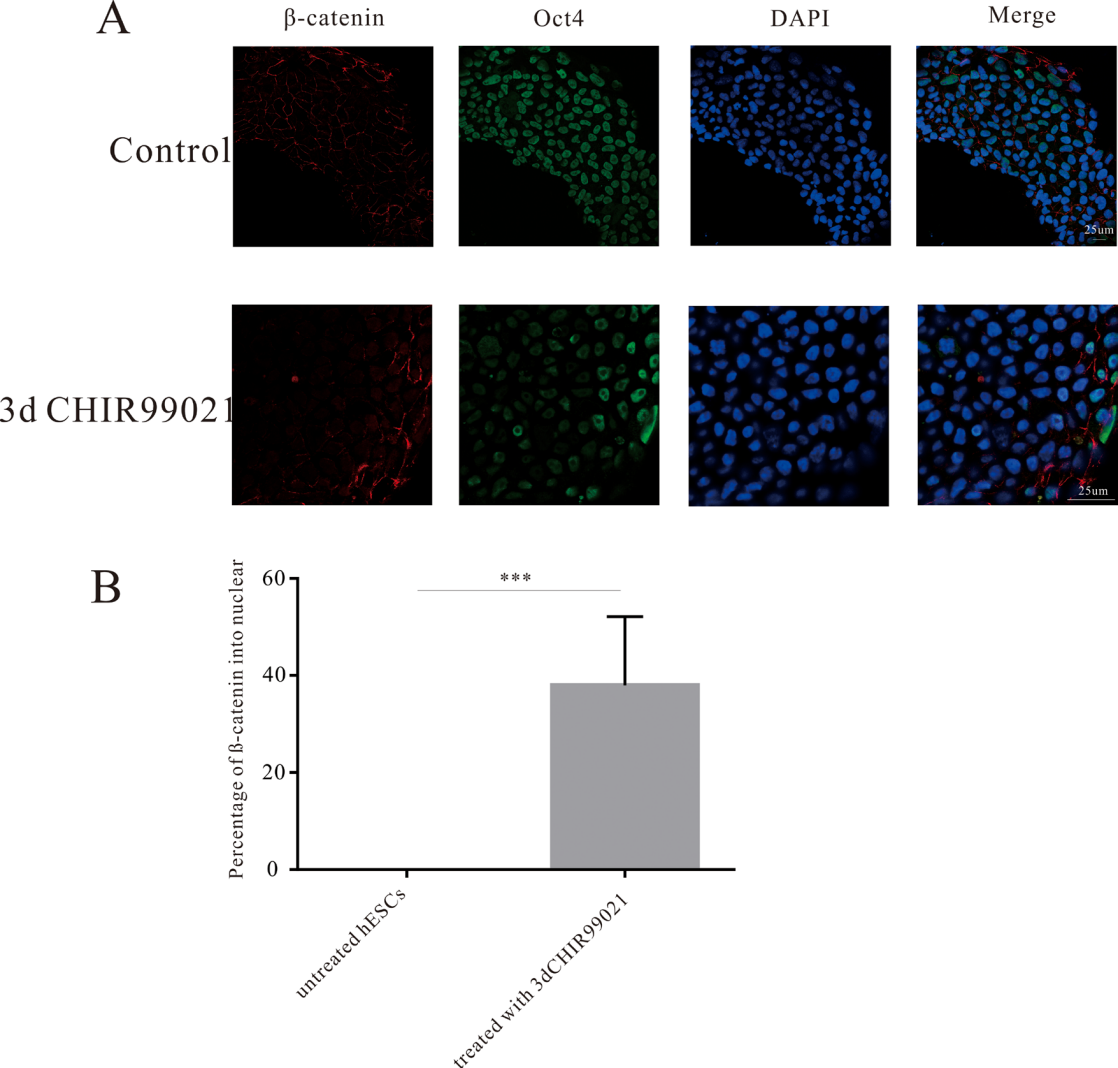
CHIR99021 activated Wnt signaling pathway via a long-term manner (**A**) Immunofluorescence analysis showing the expression of OCT4, DAZL, and β-catenin after the treatment of 3 d CHIR99021. Scale bars, 25 μm. (**B**) Statistical analysis of the percentage of β-catenin in the nuclei. All data are presented as means ± SEM. *p < 0.05, **p < 0.01,***p < 0.001.

It is reported that long time activation of Wnt signaling pathway can induce the differentiation of hESCs [34]. Consistent with this study, we treated on the hESCs with CHIR99021 for 3 d. Compared to the undifferentiated hESCs, the immunofluorescence results (Figure 4A) clearly showed that increased β-catenin had been obviously translocated from the cytoplasm into the nucleus. In addition, these percentage of β-catenin accounted for 40% (Figure 4B).

**Figure 5:**
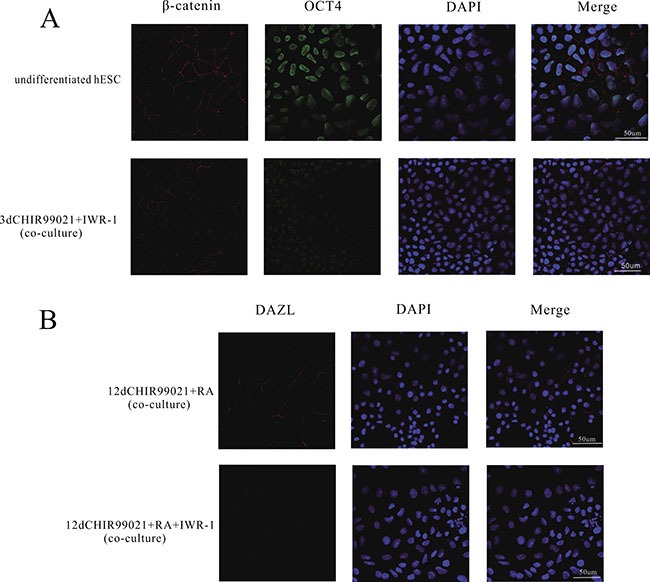
IWR-1 inhibited the accumulation of β-catenin in the nuclei and decreased the number of DAZL positive cells (**A**) Immunofluorescence straining of β-catenin and OCT4 in 3 d CHIR99021 plus IWR-1 co-culture group and undifferentiated group. Scale bars, 50 μm. (**B**) Immunofluorescence straining of DAZL in 12 d CHIR99021, IWR-1 and RA co-culture group and undifferentiated group. Scale bars, 50 μm.

IWR-1 is known to suppress the canonical Wnt signaling pathway through stabilizing the Axin and the β-catenin destruction complex [35]. To verify the role of Wnt signaling pathway in CHIR99021 mediated effects, we added IWR-1 into the 3 d CHIR99021 treatment and CHIR99021 combined with RA co-culture. As shown in Figure 5A, compared with the undifferentiated hESCs, 3 d CHIR99021 and IWR-1 co-culture treatment not only markedly reduced expression of Oct4, but also prevented β-catenin from translocating into the nucleus. Therefore, the downstream of Wnt signaling pathway cannot be initiated.

Compared to the CHIR99021 and RA co-culture treatment, the DAZL protein was not induced in the CHIR99021, IWR-1 and RA co-culture group (Figure 5B) illustrating that Wnt signaling pathway plays a very important role in inducing PGCs. These results further prove our hypothesis that the activation of the Wnt signaling pathway by CHIR99021 plays a decisive role in the whole process.

## DISCUSSION

In this study, we showed that CHIR99021 combined with RA induced PGCs from hESCs. At the beginning, the 12 d CHIR99021 and RA co-culture and the 3 d CHIR99021 plus 9 d RA groups were found to promote the expression of DAZL, which is a germ cell specific RNA binding protein and plays an indispensible role in germ cell development in mammals both *in vivo* and *in vitro* [11, 32, 36, 37]. Subsequently, western blotting, the RT-qPCR and the flow cytometry provided strong evidence to support the fact that these two small molecules do have an effect on the induction of PGCs. SCP3 straining and FISH analysis confirmed that meiosis was initiated and putative haploid cells were produced. From a perspective of reproductive medicine, we provide a simple and feasible method for generating PGCs *in vitro* and solve the practical and ethical difficulties associated with obtaining human tissue in early development. This method may serve as an important tool for further exploring the development of germ cells *in vitro* and deciphering the pathological mechanisms of male infertility.

RA, a metabolite of vitamin A (retinol), mediates the functions of vitamin A and is required for growth and development. There is a general consensus that dietary retinol or vitamin A is needed for normal spermatogenesis [38]. Recently, researchers have found that RA can not only trigger spermatogonial differentiation through Oct4 and PLZF [39], but also play an indispensible role in promoting the translation of the Kit mRNAs via activating the PI3K/AKT/mTOR signaling pathway [30]. As an important inducible factor, RA was reported to participate in inducing the embryoid body (EB) derived from human/mouse embryonic stem cells to differentiate toward germ cells with or without other exogenous factors *in vivo* [40–43]. However, we used directed differentiation system with RA, so it may explain why single RA-treatment on hESCs differentiation could not induce PGCLCs when this culture system was used.

As described above, CHIR99021 can activate the Wnt signaling pathway and promote free cytoplasmic β-catenin to accumulate and even exceed the binding capacity of E-cadherin, leading to the translocation of β-catenin into the nucleus and the regulation of the downstream LEF/TCF transcriptional complexes [27–29, 34]. Meanwhile, much progress has been reported recently about the role of CHIR99021 in inducing hESCs/iPSCs into different cell types, including cells expressing makers of the mesendoderm, the nephrogenic intermediate mesoderm and cardiomyocytes [44–47]. Remarkably, a recent genome-wide expression analysis showed that 6-bromoindirubin-3′-oxime (BIO, another GSK3 inhibitor) can significantly upregulate the reproductive development process, gland development and the development of primary sexual characteristics in mESCs [48]. However, the role of CHIR99021 in inducing hESCs into PGCs is much less known. Here, we found that CHIR99021 combined with RA can promote hESCs direct differentiation into PGCs. Similar to the previous studies, our results showed that the Wnt signaling pathway was also activated by a 3 d treatment with GSK3 inhibitor, along with β-catenin being noticeably translocated into the nucleus.

**Figure 6:**
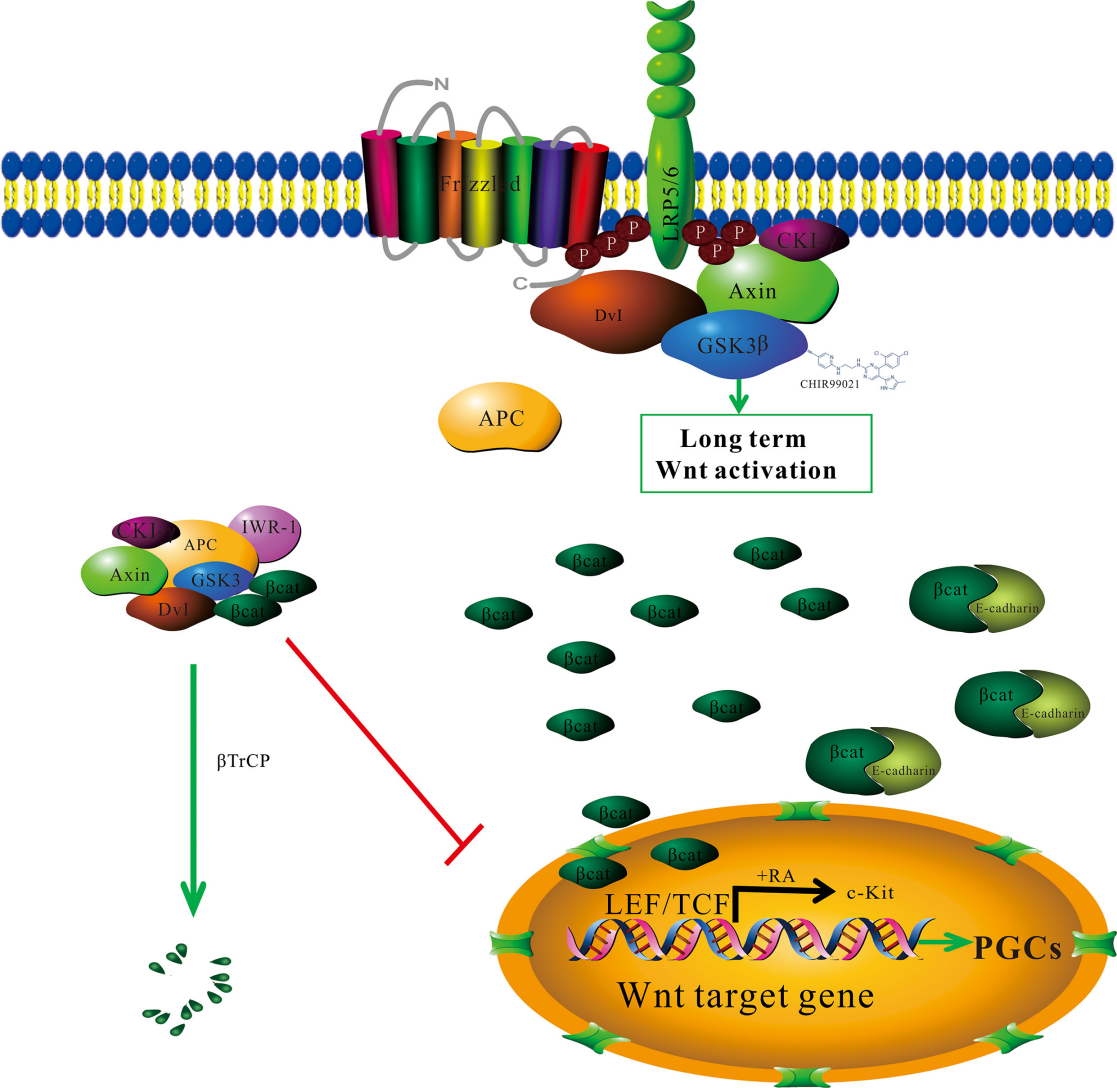
The proposed working model

To further explore the contribution of CHIR99021 to differentiation, we chose a small-molecule inhibitor of the Wnt signaling pathway, IWR-1. It induces Axin2 protein levels and promotes β-catenin phosphorylation by stabilizing Axin-scaffolded destruction complexes. After blockade the Wnt signaling pathway, we can not observe that the translocation of β-catenin into nucleus and the expression of DAZL protein in the optimum induced protocol. Thus, we recognized that the 3 d CHIR99021 following 9 d RA may work as follows: the activated Wnt signaling pathway by CHIR99021 firstly causes the translocation of β-catenin into the nucleus; then, RA induces the emergence of PGCs by the up-regulation of c-Kit. Moreover, the co-culture with CHIR99021 and RA may explain that the Wnt signaling pathway was constitutively activated by CHIR99021 even past the first 3 d (during the remaining 9 d), and RA acts in the same function as described above (Figure 6). In the future, it would be very interesting to test how to induce mature sperm cells and to improve the efficiency of induced sperm cells.

In summary, we provide a simple and feasible method for the induction of hESCs to differentiate into PGCs. In brief, CHIR99021 promoted the β-catenin translocating into the nucleus and activated the Wnt signaling pathway. Then, RA induced the expression of c-Kit and further induced the hESCs to differentiate into PGCs. In the future, “the PGCs” induced by our method may be used as a model for the screening of drugs and studying of male infertility related disease.

## MATERIALS AND METHODS

### hESCs lines and culture media

The human ESC lines Zh2 and Zh22 (ZZU-hESC-2 and ZZU-hESC-22, ZZU: Zhengzhou University) were obtained from the University of Zhengzhou. Both lines were established by isolating the inner cell mass of *in vitro* fertilized 3AA and 5AA blastocysts provided by Embryonic Stem Cell Laboratory of the Reproductive Medical Center of the First Affiliated Hospital of Zhengzhou University as described previously [49]. Both lines had male Karyotypes with 46, XY. The two hESCs lines were routinely grown in 35 mm dishes on inactivated primary mouse embryo fibroblasts (PMEF) and cultured by a hESCs growth medium consisting of Dulbecco's modified Eagle's medium (DMEM)-Ham's F-12 medium (F12) supplemented with 20% Knockout serum replacement (KSR), 1% nonessential amino acids (NEAA), 2 mM L-glutamine, 0.1 mM β-mercaptoethanol, and 10 ng/ml of basic fibroblast growth factor (all from GIBCO, USA). In order to induce the PGCs, hESCs were transferred into feeder-free conditions with conditioned medium obtained by incubating culture medium with MEFs for 24 h supplemented with 10 ng/ml bFGF. After passaging for 2 generations, the hESCs were divided into 5 groups and treated as follows: i: single 8 μM CHIR99021 for 12 d; ii: single 2 μM RA for 12 d; iii: combined with both 8 μM CHIR99021 and 2 μM RA for 12 d; iv: 8 μM CHIR99021 was added in the first three days, and then single 2 μM RA was used for the next 9 d; v: adding 2 μM RA for the first three days and then single 8 μM CHIR99021 for the next 9 d. Both CHIR99021 and RA mentioned above were diluted in hESCs medium without bFGF (see Figure 1A). Moreover, 5 μM IWR-1 was used in this study.

### Immunofluorescence

The cells were washed twice with phosphate buffer solution (PBS) and then fixed with 4% paraformaldehyde for 15 min at room temperature. After washing twice with PBS, the cells were subsequently permeabilized with 0.1% triton X-100 for 10 min and then blocked with 5% bovine serum albumin (BSA) for 30 min. After the blockade, the cells were stained with the following antibodies: anti-DAZL (ab34139; abcam), anti-Oct4 (MAB4419A4; Millipore) and anti-β-catenin (#9562; Cell Signaling Technology) overnight at 4°C. After washing twice with PBS and incubating with 5% BSA for 30 min, the cells were incubated with the second antibodies (Alexa Fluor 488-conjugated goat anti-mouse IgG, Alexa Fluor 574-conjugated goat anti-rabbit IgG, Invitrogen) for 1 h at room temperature. After washing, the cells were incubated in diamibino-phenyl-indole (DAPI, 14016–01, BIODEE) for 30 min. Finally, the cells were mounted on slides and examined by using a laser scanning confocal microscope (Leica SP5). The images were analyzed by using the FIJI software.

### Western blotting

After rinsing with cold PBS, the cells were harvested and lysed on ice with RIPA buffer containing phenylmethanesulfonyl fluoride for 30 min. Then, the liquid was transferred into 1.5 mL tubes and centrifuged at 12, 000 g for 25 min to obtain the supernatant. The protein concentrations were determined by using the BCA assay. Before loading on gels, the cell lysates were heated at 90°C for 5 min in 2X sample loading buffer containing 5% β-mercaptoethanol. Five mg proteins were resolved on a 10% SDS gel. The resolved proteins were transferred to PVDF membrane (Millipore) at 300 mA for 1.5 h. The membranes were blocked for 1 h with Tris-buffered saline-Tween 20 (TBST) containing 5% skim milk powder at room temperature. Then, the PVDF membranes were incubated overnight with anti-DDX4 (#8827; 1:1000, Cell Signaling Technology), anti-β-catenin (#9562; 1:1000, Cell Signaling Technology), anti-acrosin (sc-46284; 1:200, Santa Cruz Biotechnology) and anti-β-actin (60008-1-AP; 1:20000, Proteintech) at 4°C. After washing and incubating with the secondary antibodies, the final detection was performed using enhanced chemiluminescence detection solutions 1 and 2 (1:1) (ECL, Millipore). The protein-signal densities were normalized to the corresponding β-actin-signaling densities.

### Fluorescent *in situ* hybridization

For FISH analysis, the cells were harvested and dissociated into single cells with 0.25% trypsin and then washed in PBS. Cell pellets were treated with hypotonic solution (0.075 M KCL) for 25 min at 37°C, then fixed twice with Carnoy's fixative (1:3 acetic acid: methanol) for 20 min and then cell pellets were dropped onto the slides and dried at 56°C in drying box for 2 h. Then the slides were washed (Sangon, Shanghai, China) in 2X SSC for 10 min, following by a dehydrated procedure in 80%, 90% and 100% ethanol series for 3 min each. FISH probes (Chr16, Chr 22, GP, China) were denatured on slides at 76°C for 6 min on a hot plate and further hybridized at 42°C in a moist chamber for 16 h. In the following step, the slides were washed with 0.3%NP-40/0.4X SSC for 2 min, 0.1%NP-40/2X SSC for 30 sec and 70% ethanol for 3 min, respectively. Finally, the slides were strained with DAPI.

### Immunofluorescence staining of synaptonemal complexes

For the straining of synaptonemal complexes, cells were lysed by a hypotonic solution for 30 min at room temperature, following submerged in 1% PFA on glass slides overnight at room temperature. The slides were washed with ddH_2_O for 4 min, then blocked with ADB buffer (diluting 1% donkey serum, 0.3% BSA and 0.01% triton X-100 in PBS buffer) at room temperature for 30 min and incubated with primary antibody against human SCP3 (mouse polyclonal, 1:200, Abcam) overnight at 37°C. The slides were further washed with 1X TBS 10 min 3 times following blocked with ADB buffer for 6 h at 4°C, and then washed with 1X TBS 10 min 3 times again and incubated with goat anti-mouse FITC-conjugated (1:120, Santa) secondary antibodies at 37°C for 1.5 h and mounted as described above.

### Real-time RT-qPCR

Total RNA was extracted from the cells with the TRIZOL RNA purification system (Invitrogen). According to the manufacturer's instructions (TAKARA), cDNA was generated from the mRNA (1 μg) using the PrimeScript™ RT reagent Kit with gDNA Eraser (RR047A, TAKARA). Quantitative real-time RT-qPCR was performed using EvaGreen 2X qPCR Mix (master Mix-S, abm) in a Roter-Gene Q-QIAGEN machine RT-PCR with the following profile: 95°C for 10 min, followed by 40 cycles of 95°C for 15 s and 60°C for 60 s. The amount of Evagreen signal was recorded at the end of each cycle. The levels of target mRNAs analyzed by RT-qPCR were normalized to the level of GAPDH. Detailed information regarding the primer pairs used in this experiment was shown in Table 1. The data presented are the average of at least 3 independent experiments.

**Table 1:** List of Q-PCR primer sequences used in this study (Related to experimental procedures)

Genes	Forward	Reverse
DDX4	CCATTGTGGATGTATTATCTCGCTTA	GCCTCTGGGCGGAATTTT
Blimp-1	AACTTCTTGTGTGGTATTGTCGG	CAGTGCTCGGTTGCTTTAGAC
Nanos	GACGCTTCTGCCCACTTACTG	GTCCTGTGTCTTCGCCTTGTC
TFAP2C	TCAGTCCCTGGAAGATTGTCG	CCAGTAACGAGGCATTTAAGCA
GAPDH	GAAATCCCATCACCATCTTCCAGG	GAGCCCCAGCCTTCTCCATG

### Flow cytometry

For flow cytometric analysis, the cells were harvested and dissociated into single cells with 0.25% trypsin and then washed in PBS plus 0.5% BSA twice. After washing, the cells were stained with antibodies to CXCR4 (FAB170P; R&D system), c-Kit (FAB332A; R&D system) and DAZL (ab34139; abcam) for 40 min at 4°C. After washing by PBS twice, the cell pellet was re-suspended in 300 μl PBS for the final flow cytometric analysis of CXCR4 and c-Kit. Besides, the second antibody (ZF-0511, 1:400, Alexa Fluor 488-conjugated goat anti-rabbit IgG, ZSGB-BIO) was added into DAZL treatment for 40 min at 4°C. After that, the cells were washed twice with PBS and re-suspended in 300 μl of PBS for the final flow cytometric analysis. As a control for the analysis, the undifferentiated cells in a separate tube were treated with a mouse IgG_1_ APC-conjugated antibody (IC002A; R&D system) and a mouse IgG_1_ isotype control-PE (IC002P; R&D system) for c-Kit and CXCR4, respectively. For DAZL, the undifferentiated cells were only strained with the second antibody. Finally, cells analysis was performed on the Becton-Dickinson FACS Calibur platform.

### Statistical analysis

Results are expressed as mean ± Standard error of arithmetic mean (SEM). Statistical significance was determined using a Student's *t-test* and one-way ANOVA. Results were considered significant when *p* < 0.05.
